# Echoes of the Brain: Local-Scale Representation of Whole-Brain Functional Networks within Transmodal Cortex

**DOI:** 10.1177/1073858415585730

**Published:** 2015-10

**Authors:** Rodrigo M. Braga, Robert Leech

**Affiliations:** 1 The Computational, Cognitive and Clinical Neuroimaging Laboratory, Division of Brain Sciences, Hammersmith Hospital Campus, Imperial College London, London, UK; 2 Center for Brain Science, Harvard University, Cambridge, MA, USA; 3 Athinoula A. Martinos Center for Biomedical Imaging, Department of Radiology, Massachusetts General Hospital & Harvard Medical School, Charlestown, MA, USA

**Keywords:** transmodal cortex, multimodal, association, integration, networks, hubs, intrinsic connectivity networks, neural dynamics

## Abstract

Transmodal (nonsensory-specific) regions sit at the confluence of different information streams, and play an important role in cognition. These regions are thought to receive and integrate information from multiple functional networks. However, little is known about (1) how transmodal cortices are functionally organized and (2) how this organization might facilitate information processing. In this article, we discuss recent findings that transmodal cortices contain a detailed local functional architecture of adjacent and partially overlapping subregions. These subregions show relative specializations, and contain traces or “echoes” of the activity of different large-scale intrinsic connectivity networks. We propose that this finer-grained organization can (1) explain how the same transmodal region can play a role in multiple tasks and cognitive disorders, (2) provide a mechanism by which different types of signals can be simultaneously segregated and integrated within transmodal regions, and (3) enhance current network- and node-level models of brain function, by showing that non-stationary functional connectivity patterns may be a result of dynamic shifts in subnodal signals. Finally, we propose that LFA may have an important role in regulating neural dynamics and facilitating balanced activity across the cortex to enable efficient and flexible high-level cognition.

## Introduction

The human neocortex has expanded asymmetrically during its evolution (Hill and others 2010; Krubitzer 2007). “Unimodal” areas, which predominantly receive input from a single sensory modality (such as vision or audition), occupy a smaller proportion of total brain volume in humans than many mammals (Krubitzer 2007). On the other hand, “transmodal” regions have undergone a drastic expansion (Hill and others 2010). The term *transmodal* was proposed by Mesulam (1998) to refer to cortical regions where task-driven increases in activation are not specific to any single sensory modality, and also produce disparate, non-specific symptoms when lesioned. Key transmodal areas include the association cortices (also known as “heteromodal” or “multimodal” areas), and higher order cognitive networks, including the frontoparietal, salience, and default mode (DMN) networks. In line with their transmodal role, these regions are implicated in a range of higher order cognitive abilities (e.g., Buckner and others 2009; Laird and others 2013; Smith and others 2009). Normal and aberrant activity within transmodal cortex is indicative of individual differences in cognitive ability (Duncan and others 2000; Hampshire and others 2012; Mueller and others 2013; Seeley and others 2007) and mental health (Buckner and others 2009; Menon 2003; Mueller and others 2013). These findings point to transmodal cortices as playing an important role in enabling the complex cognitive processes available to humans.

## The Importance of Intermediates

**Figure 1.**
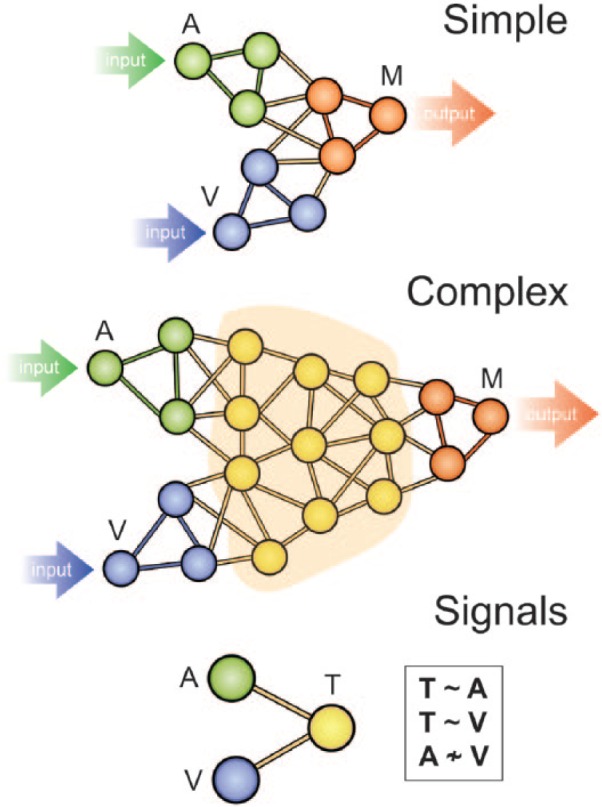
Intermediate structures (yellow) between sensory input (green, blue) and motor output (red) pathways allow more flexible control of behavior as more regions can determine the output of motor cortices (M). The convergence of auditory (A) and visual (V) signals at intermediate structures means that the activity of a transmodal region (T) should be correlated with the signal from both A and V, even if A and V are not correlated with each other (Xu and others 2013). The signal from A and V should therefore be detectable in region T by decomposing the signal from T into its constituent components. Adapted from Mesulam (1998).

Transmodal regions may be thought of as performing an *intermediary* role: They interconnect separate unimodal sensory systems with other transmodal as well as motor output systems (Fig. 1; Mesulam 1998). Theoretically, for an organism to possess a limited repertoire of responses to its environment, simple connection pathways between sensory and motor neurons are sufficient (Fig. 1). Although simple systems can produce fast and efficient responses (e.g., Krasne and Wine 1984), they also lead to automatic, inflexible behaviors that are undertaken even when the outcome may be detrimental (Mesulam 1998). A classic example is the frog visuomotor system (Ingle 1970), which has a small number of synapses between retinal and motor neurons. Presentation of a visual stimulus within a certain range of features (e.g., size, contrast, motion) will elicit prey-catching behavior (tongue-snapping) regardless of whether the stimulus is edible (Ingle 1968). If the frog-eye is rotated 180° and allowed to re-innervate the optic tectum, the frog will lick the ground whenever a stimulus is presented overhead (Sperry 1945). This misguided behavior is persistent and remains inflexible even after extensive training (Sperry 1945).

An analogous situation can be induced in humans using inversion goggles, which rotate the visual input by 180° (Kohler 1963). Although initially disoriented, subjects will grow accustomed to the new orientation and be able to perform complex behaviors (e.g., reaching, writing, cycling). In contrast to the relatively “simple” visuomotor wiring found in amphibians, humans have an expanded set of intermediary regions between visual and motor cortices (Fig. 1C; Mesulam 1998). At these intermediate regions, information from different sensory sources converge (Sepulcre and others 2013) a process that is likely to be necessary for guiding more complex behavioral responses. For example, by integrating multiple sources the organism may be able to flexibly select or influence which information streams are allowed to guide behavioral output (Fig. 1), consistent with the notion of selective attention (Corbetta and Shulman 2002; Downar and others 2000).

## Converging Signals

If the hierarchical organization proposed by Mesulam (1998; Fig. 1) is correct, hypotheses about the neural activity in integrative regions can be formed and tested. For example, if the neural activity from two brain networks, A and V, converge within a transmodal region T (Fig. 1), the activity of T may be partially correlated with both A and V, even if A and V are not correlated with each other (Xu and others 2013). This means that the signal obtained from T (e.g., using functional magnetic resonance imaging [fMRI]) can be expected to contain a mixture of the signals from A and V. However, the mean signal from T may not provide a good correlation with A or V, as T contains multiple signals which, when averaged together, may have both constructive and deleterious effects. Instead, multivariate source separation techniques such as independent component analysis (ICA) can be used to separate mixed signals into their constituent components (Bell and Sejnowski 1995). Multivariate techniques could therefore be used for probing the activity structure of transmodal regions and detecting the convergence of information streams.

## Evidence for Convergence

Several lines of evidence have emerged to suggest that transmodal cortices are indeed sites where neural inputs converge. Histological studies have identified sites where neural projections from primary sensory regions converge within transmodal cortex (Goldman-Rakic 1988; Jones and Powell 1970; Pandya and Kuypers 1969). Diffusion tractography has been used in humans to show that transmodal regions contain the highest number of connections with widespread systems (Hagmann and others 2008), including other highly connected regions (van den Heuvel and Sporns 2011), an organization that might be expected for an integrative system (Fig. 1). Functionally, task fMRI studies using stimuli of different sensory modalities also implicate certain regions as being modality invariant or “amodal” (Beauchamp 2005; Corbetta and Shulman 2002; Downar and others 2000; Langner and others 2011). In particular, the lateral occipito-temporal junction and posterior parietal lobe have been consistently implicated in the integration of vision, touch, and audition (e.g., Beauchamp 2005; Calvert and others 2001, Driver and Noesselt 2008). Similarly, transmodal regions of the prefrontal cortex have been associated with diverse functions such as multimodal integration, spatial processing, response-inhibition and short-term memory (Cabeza and Nyberg 2000; Fuster 1980; Jacobsen 1936; Rosenkilde 1979; Stuss and Benson 1984). More generally, when brain activity is viewed as being composed of “intrinsic connectivity networks” (ICNs, also known as “resting state networks” or “functional networks”; Bressler 1995; Cordes and others 2000; Horwitz and others 1984; Smith and others 2009; Yeo and others 2011) of temporally coactivating regions, often multiple cognitive tasks are implicated on the same set of ICNs (Buckner and others 2008; Laird and others 2013).

Functional MRI functional connectivity (FC; Biswal and others 1995) has also been used to show that transmodal cortices are functionally connected to widespread cortical regions (Buckner and others 2009; Bullmore and Sporns 2009; Mesulam 1998; Sporns and others 2007). Importantly, Sepulcre and others (2013) showed that, when assessed in a stepwise manner, the FC of primary visual, auditory, and somatosensory cortices all converge in transmodal regions. Default mode regions were found to sit at the top of this hierarchy (Buckner and others 2009; Goldman-Rakic 1988; Sepulcre and others 2013). The organization of brain activity into ICNs also seems to follow a hierarchical structure (Doucet and others 2011; Meunier and others 2009). Transmodal cortices also communicate with a higher number of networks and show higher functional heterogeneity than other regions (Andrews-Hanna and others 2010; Doucet and others 2011; Leech and others 2012; Mueller and others 2013; Sepulcre and others 2013; Sporns and others 2007), which further supports their role as sites of convergence.

## Local Functional Architecture Supporting Convergence

Although the evidence for convergence at transmodal regions is strong, much less is known about how transmodal cortices are organized to support this convergence. In particular, little is known about how transmodal cortices are organized at the local scale; whether all signals converge on the same functionally homogeneous region, or whether there are important functional subdivisions, or a “local functional architecture” (LFA), within transmodal cortex.

The first possibility is that transmodal regions communicate with many networks through a functionally homogeneous region within which synaptic connections with distributed systems are evenly dispersed. For example, there is evidence that the activity from a single voxel can be attributed to more than one ICN at a time (Yeo and others 2014). In addition, neighboring neurons can sometimes display reliably different time courses of activation for the same task, suggesting the overlap of signals is present even at microscopic distances (Chafee and Goldman-Rakic 1998; Fuster 2009; Verduzco-Flores and others 2009). Although feasible, a homogeneous organization would place unsustainably high demands on the local vasculature, as the modulation of any converging signal would require metabolic resources to be supplied to the same area of cortex continuously. A possible solution to this would be to ensure that the transmodal region only communicates with a subset of distal regions at any one point in time, such that over time the region’s FC would shift to different networks over time. There is evidence that transmodal regions do display dynamic shifts of FC when assessed at the network level (de Pasquale and others 2012; Smith and others 2012, Calhoun and others 2014); although the presence of spatially segregated subregions at finer spatial resolutions would still be compatible with these findings.

An alternative possibility is that, rather than being distributed homogeneously, the multiple signals which converge on transmodal regions are spatially organized into an LFA. Such an organization could reduce the metabolic demands made by any one cortical area, and potentially allow more efficient neurovascular coupling. In addition, an LFA could allow for subregional specialization of neural computational and facilitate the integration of the locally distributed signals. As an example, consider the posterior cingulate cortex (PCC). The PCC is a central node of the DMN (Shulman and others 1997). The DMN itself can be divided into subregions based on intrinsic connectivity and task activation differences (Andrews-Hanna and others 2010; Leech and others 2012; Margulies and others 2009). Within the functional network framework, the PCC and precuneus have typically been considered a single node of the DMN, with the whole region sharing a considerable amount of signal. Perhaps because of this shared signal, it has been difficult to probe the LFA-level subnodal structure within transmodal regions like the PCC. However, from a cytoarchitectonic perspective subdivisions within the PCC have been proposed (Brodmann 1910; Vogt and others 2006). Furthermore, subregions of the PCC have been shown to react differently under different task conditions (e.g., Leech and others 2011). Therefore, a deeper exploration of the functional organization of the PCC at this LFA level is needed. Particularly, exploring how the subdivisions of transmodal nodes, if present, interact over time could lead to a better understanding of how transmodal cortices support the convergence and integration of multiple inputs.

## “Echoes” of the Brain

**Figure 2.**
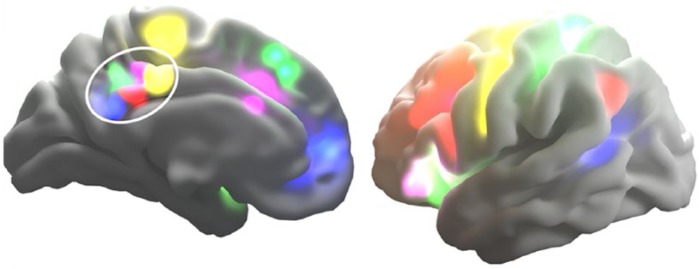
Subregions of posterior cingulate cortex (PCC; within the white ellipse) and how each is functionally connected with (“echoes”) different whole brain intrinsic connectivity networks (shown in different colors).

In a recent article, we showed that the fMRI signal from the PCC can be decomposed into multiple meaningful subsignals (Fig. 2; Leech and others 2012). We used a spatial ICA to split the PCC into subregions, and then used multiple linear regression to extract partialled time courses from each of these subregions simultaneously. The FC of these time courses with the rest of the brain was assessed using a second regression (dual regression), to reveal the whole-brain FC pattern for each PCC subregion defined. This allowed the PCC to display multiple patterns of FC, and allowed us to probe the origin of the different signals which converge on the PCC.

We found that these component signals, obtained from adjacent parts of the PCC, produced strikingly different FC patterns. Many of the resulting FC maps resembled the well-characterized whole-brain ICNs that are typically obtained from whole-brain clustering techniques such as ICA (Smith and others 2009), *k*-means clustering (Yeo and others 2011) or univariate seed-based FC analyses. This provided evidence that the PCC was in constant communication with many different ICNs, which suggests that the PCC is a site of convergence of signals from different networks, and is therefore well placed to modulate and integrate the information from many ICNs. The identification of functional subdivisions suggests that the PCC mediates this convergence through a complex LFA of component subregions. In a sense, the PCC was found to capture much of the complexity that is present (at a coarse resolution) in the whole-brain itself: it contains a brain-network-topic map.

Importantly, when the FC of the PCC is assessed using conventional univariate approaches, which take a single, averaged time course to represent many voxels, the DMN signal typically dominates the PCC’s FC structure (although some differences are still observable; Margulies and others 2009). This means that the existence of multiple signals remains hidden (see Leech and others 2012). The existence of these mixed signals which represent large-scale ICNs led us to coin the term *echoes of the brain*. Importantly, the approach by Leech and others (2012) and Braga and others (2013), used time courses that covaried other signals found in the PCC. As such, the covaried signals represent *relative specializations* within the PCC, which are uncovered only when the shared signal is controlled for. In contrast, when the shared signal is not covaried out, subregions of the PCC are often reliably clustered together as a single node of the DMN. These findings suggested to us that the normal decomposition of brain activity into nodes and networks of nodes may be enhanced by considering that a subset of the nodes contain “subnodes”; relatively specialized subdivisions that support the intermediate role of transmodal cortices.

## Spatial Distribution of Echoes

The subsignals we identified showed a consistent spatial organization across subjects. In general, the subregions of the PCC were found be contiguous, with a broadly bilateral organization. The core of each subregion displays a relative specialization for a given ICN while being in close proximity to other regions. In addition, the subsignals we observed were not just neighboring but also partially overlapping. This functional organization could allow for the simultaneous segregation and integration of neural signals; two features that are thought to be important for information processing (Tognoli and Kelso 2014). The overlap of functional networks has been observed using other whole-brain multivariate analysis approaches (Geranmayeh and others 2014; Xu and others 2015; Yeo and others 2014). Consistent with transmodal regions supporting multiple signals, the cytoarchitectonic complexity has been shown to correspond to measures such as the degree of rich club organization, and be higher in many transmodal brain regions (Scholtens and others 2014). The existence of intermixed signals at overlapping subregions, within a resolution smaller than that of an individual voxel, would suggest an organization that potentially allows very highly controlled and rapid interactions between signals. At present, it is difficult to precisely map out the structure of these subregions due to the low signal-to-noise ratio of fMRI, which necessitates spatial smoothing and averaging across subjects (Hopfinger and others 2000). Advances in individual-subject fMRI (e.g., using 7T MRI) should allow for a more detailed exploration of LFA in transmodal cortices.

**Figure 3.**
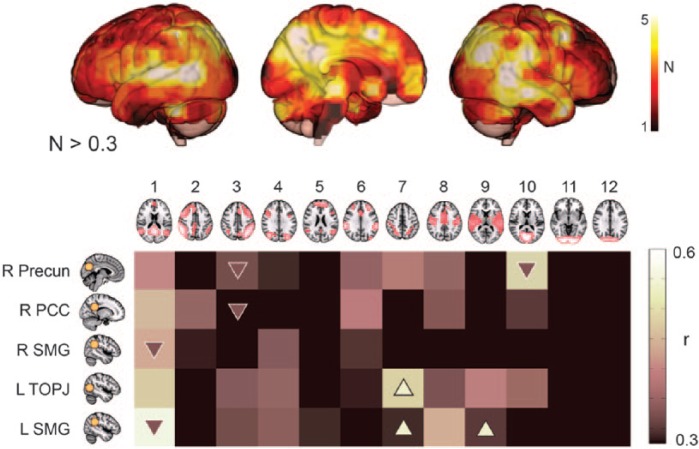
Different echoes from different transmodal regions. (Top panel) Some, but not all, regions of the cortex were found to contain multiple hidden signals (or “echoes”) relating to whole-brain intrinsic connectivity networks (ICNs: numbers 1-12; N: number of ICNs detected at each region above a spatial correlation threshold of 0.3). In general, few signals were detected in unimodal sensory areas (cold colors) compared to transmodal areas (hot colors). Known transmodal regions such as the precuneus (Precun), posterior cingulate cortex (PCC), temporo-occipito-parietal junction (TOPJ), and left (L) and right (R) supramarginal gyrus (SMG) were found to contain many hidden signals. (Bottom panel) Each of these transmodal centers was found to contain signals from different combinations of whole-brain networks, suggesting that each center mediates the convergence of information from different sources. The different signals also showed differential task-modulation during an attentionally engaging choice-reaction time task (arrows in matrix). This suggests that the activity of transmodal cortex could be driven by different echo subregions during different task contexts, which could explain why similar transmodal recruitment is observed across many different tasks. Adapted from Braga and others (2013).

In a follow-up study, we used a searchlight approach to test whether the existence of multiple subsignals could be identified in any region of the cortex, rather than just the PCC (Braga and others 2013). We found that this property was not exclusive to the PCC, with echoes also being observable in known transmodal regions such as the supramarginal gyri, right prefrontal cortex and superior parietal lobe, and medial dorsal cingulate and superior frontal cortices. Most of these transmodal regions contained a subregion that was connected to the DMN. They also contained subregions connected to other whole-brain ICNs, but different combinations of ICNs were observed in each transmodal region (Fig. 3). This organization allows a certain amount of redundancy, in that the information from a given pair of networks may be represented in multiple transmodal centers. However, it also suggests that each transmodal region may play a unique role in integrating different sources of information. In agreement with Mesulam’s (1998) hierarchical cortical organization, unimodal sensory regions showed little evidence of containing multiple signals of ICNs. However, it is worth pointing out that this does not mean that these regions do not contain complex signals. A unimodal visual region may contain multiple signals related to vision. In contrast, our analysis specifically probed for the presence of signals from whole-brain ICN.

## Possible Explanations for Echoes (See Also Illustrative Video in Supplementary Material)

### Integration of Signals

The most straightforward interpretation for the existence of echoes is that they are a feature of how transmodal brain regions operate to enable efficient neural information processing. The echoes are consistent with Mesulam’s interpretation that the heterogeneous connectivity of transmodal cortices allows the integration of information from multiple sources in order to facilitate flexible cognition. Within these identified transmodal regions, we observed signals not just from multiple sensorimotor and heteromodal sources, but also from other transmodal brain regions. This feature is consistent with the “rich-club” organization observed in functional and structural networks by van den Heuvel and Sporns (2011), where highly connected nodes are connected to other highly connected nodes. The shared signal across a transmodal node may reflect local communication and, presumably, functional coherence between subregions. The presence of relative specialization in each subnode region, which is embedded in the dominant shared signal, suggests that transmodal nodes should be thought of as a loose coalition of subregions, rather than a single homogeneous unit.

**Figure 4.**
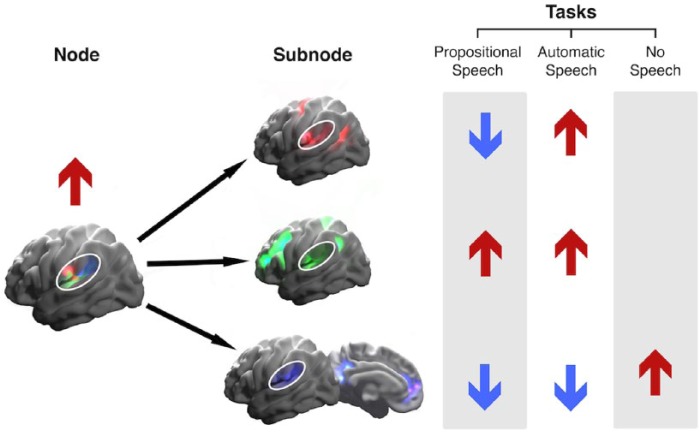
An example of local functional architecture extracted from the posterior sylvian fissure, known to be an important convergence zone for different sensorimotor signals and important for speech production (Simmonds and others 2014). The activity of the whole region may relate to the summed activity of the different subregions (shown in different colors on the left). Different task conditions (e.g., propositional speech production or automatic speech or a no-task condition) involve different whole-brain intrinsic connectivity networks, which are each associated with an increase in activity in specific peri-sylvian subregions (on the right). We hypothesize that activity then propagates to adjacent subregions, facilitating integration of information. Therefore, overall activity in the region, viewed at a coarser scale, may reflect very different functional roles mediated by smaller subregions. This overall activity could have a net positive or negative summation, or could balance out. For example, in this case there may be an overall positive level of activity during automatic speech, when parietal and temporal subregions are both positively activated even though ther is a negative contribution from the DMN subregion.

To understand how the integration of signals occurs, we need to examine how specific subsignals are modulated by different task conditions. For example, the posterior sylvian fissure (overlapping with Wernicke’s area) was found to contain subsignals connected with somatosensory and auditory networks that overlap within posterior peri-sylvian regions (Simmonds and others 2014, see Fig. 4). These subsignals were differentially activated during different speech production conditions (e.g., propositional speech production versus simple non-propositional speech such as counting), suggesting that this transmodal brain region sensitively adapts to integrate information from either somatosensory/motor or auditory sources (or both) depending on the specific task requirements (e.g. the dynamic requirement for auditory, somatosensory or motor feedback). As suggested in Figure 4, increased activity across this region could be driven by one of several subregions echoing very different whole-brain networks, with very different functional properties.

Evidence from the right lateral frontal cortex also suggests that a LFA facilitates integration of different sources of information to achieve complex cognitive skills (Erika-Florence and others 2014). During a range of tasks requiring cognitive inhibition and attentional control, subregions were found that communicate with discrete, spatially distributed frontoparietal control networks. However not all subregions displayed the same responses. In particular, a subregion that contained signals from the salience network showed increased activity with different aspects of the task (e.g., task complexity and with learning task requirements).

Finally, the DMN as a whole shows decreased activation during an attentionally engaging choice reaction time (CRT) task (Leech and others 2012). However, when the existence of multiple PCC subsignals was controlled for, only the PCC subsignals which echo the left- and right-frontoparietal networks showed robust evidence of task modulation. This result was unexpected, since it suggests that parts of the PCC that communicate with, for example, the rest of the DMN, are unaffected or much less affected by the task. We speculate that at rest (i.e., in the absence of an explicit cognitive task) the PCC is involved in communicating with much of the brain via these frontoparietal networks, possibly facilitating a broad, exploratory attentional state (Leech and Sharp 2014). When a focused state is required, the frontoparietal subsignals in the PCC reduce their activity. This may be the driving force for the reduction in the shared signal across the PCC as a whole (possibly reflecting local communication and/or coherence); however, the remaining subsignals remain unaffected in their relative specialization. This is evidenced by their unperturbed FC with other ICNs regardless of task-based modulation of the frontoparietal echo regions or the PCC as a whole.

The segregated subregions within transmodal cortex also have different timecourses, by virtue of their specialization to different ICNs. Therefore, when looked at from a coarser perspective, the larger transmodal region will show fluctuation in terms of which ICNs it is functionally connected to at any given time point. For example, an ICN such as the salience network may be functionally connected with the dorsal attention network during one cognitive state, but switch to being more connectivity to the DMN while performing a different behavioral operation (Spreng 2012). However, when considering the LFA, this switch in FC might be a consequence of the modulation of specific subnodal signals, rather than the node or network as a whole. In such a case, the average signal of the node would be weighted toward the increased subsignal, and could appear as a switch in the FC of the node.

One way that these subregions may exert their influence over distant, distributed regions is through specific frequencies that are characteristic to specific networks. There is evidence from combined fMRI/electroencephalography and magnetoencephalography studies that different ICNs may have different characteristic frequencies (e.g., Mantini and others 2007). Oscillations at specific frequencies have been proposed to help coordinate neural activity between distant brain regions (e.g., Fries 2005). Therefore, one possibility to be investigated is that different transmodal subregions have a bias toward the specific characteristic frequency of the network they “echo.” Furthermore, the LFA framework allows not only *temporal* non-stationarity of FC but also *spatial* non-stationarity. For convenience, we often (tacitly) assume that the ICNs are stable, invariant networks. While this is an attractive idea, the reality is more nuanced. The classic ICNs remold over time (Jones and others 2012) and depending on task context. For example, we and others have shown that the DMN is spatially non-stationary, changing quite substantially in terms of which frontal and parietal regions are involved in it (Leech and others 2014; Scott and others 2015; Seghier and Price 2012). This non-stationarity is consistent with the LFA we observe, as different subregions may increase or decrease their activity either spontaneously or in response to changing task requirements. The exact spatial pattern of different ICNs will therefore be context-dependent and highly fluid, as the “recruitment” of voxels within a network node will depend on the signal changes within its functionally specialized subregions. If this is true, then the *classic* brain networks that are frequently reported may be average tendencies rather than discrete entities, something that has also been suggested when considering higher temporal resolutions and network dynamics (de Pasquale and others 2012; Smith and others 2012, Calhoun and others 2014).

### Controlling Neural Dynamics

Up to now, we have considered the organization of transmodal brain regions from a functional point of view; that is, we have discussed why these brain regions are organized this way in terms of what functional benefits this might confer to cognitive and perceptual processing. However, an alternative approach is to consider more basic reasons for the organization (although these explanations are not mutually exclusive). Higher level perception and cognition in the brain is, by necessity, implemented through an electrochemical system consisting of billions of dynamically interacting neurons. The biological basis of this system constrains how information processing can be performed, and the higher order cognitive operations are constrained by how evolution has built them out of preexisting neural mechanisms.

Spontaneous intrinsic patterns of neural activity have been observed across spatial and temporal scales, and across many species. These dynamics persist across different cognitive states and persist in the face of all but the most severe damage without the system collapsing into pathological (i.e., random, flat, or saturated) dynamics. Theoretical accounts (based on self-organized criticality or stochastic resonance; Beggs and Plenz, 2003) suggest that the brain exists within an optimal dynamic range, necessary for efficient and flexible behavior (Shew and Plenz 2013). Computational models suggest that the rich-club organization of the brain facilitates these dynamics, and further that the ICNs emerge from the interaction of these dynamics through the underlying structural network topology (Deco and others 2009; Haimovici and others 2013; Senden and others 2014). We should, therefore, consider that the local-scale organization of transmodal regions—important for higher level cognition—has arisen in the context of these dynamics.

**Figure 5.**
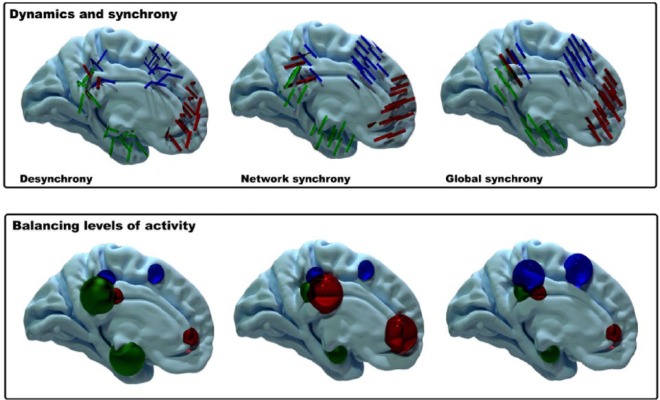
Two alternative and somewhat speculative explanations for the echoes functional organization. (Top panel) The echoes may exist to provide a convergence zone where different subregions drive each other into or out of phase, which in turn drives the whole brain into or out of synchrony, regulating neural dynamics across the brain. (Lower panel) The echoes may also allow the brain a mechanism for maintaining a balance of activity, a form of homeostasis such that increases in activation in one region are matched by decreases in another region. In this simple caricature, increased activity is matched by decreased activity in adjacent regions, but it could also operate across larger distances such as large-scale brain networks, with increases in one subregion matched by decreases in the subregion of a different brain region.

One possibility is that the LFA of transmodal regions allows them to modulate global dynamics in a controlled way. Neural dynamics change with cognitive state (e.g., Hellyer and others 2014). While the healthy, awake brain appears to operate in a rich, ceaseless dynamical regime, during a focused task the brain becomes less critical, displaying more stable and synchronized dynamics (Fagerholm and others 2015). Mechanistically, this agrees with the intuitive explanation that the brain moves from being in an exploratory (not locked into any specific input or output process) to a focused state (with a given set of processes active and a reduction in “intruding” non–task relevant dynamics). In this context, transmodal brain regions could be actively regulating the dynamic range of the system, pushing it in and out of unconstrained regimes (Hellyer and others, 2014). The “echoes” of ICNs within transmodal regions could provide a flexible way to modulate these dynamics, through local-scale interactions that couple the activation of one network with others, allowing networks to move into and out of synchrony with each (Fig. 5A) and the brain to move into and out of desynchronized or synchronized states. Recent evidence from within the right lateral frontal lobes suggests that global dynamics may be actively modulated by specific frontal subregions during very high-level cognitive tasks (i.e., a relational reasoning task; Parkin and others, in press).

### Balancing Neural Activity

A related, and possibly more outlandish idea, is that the echoes LFA is important for maintaining a local homeostatic balance in activation levels (Fig. 5). At the microscopic level, the role of local inhibitory processes linked to excitatory ones is well acknowledged (e.g., Vogels and others 2011), and is thought likely to facilitate useful computational properties and promote dynamical regimes (Magnasco and others 2009). Such a mechanism may exist to stop runaway excitation from spreading around the brain and/or to allow balanced levels of activity to be maintained. Alternatively, because of the extremely high metabolic demands of neural activity (Lord and others 2013), it is possible that any change in regional activity needs to be accompanied by some amount of regional deactivation (Leech and others 2014). Within the echoes framework, as one subregion is up-regulated (accompanying an increase in activity of its whole-brain ICN), this could be offset by spatially coupled decreases in activity in nearby subregions (Fig. 5B). Alternatively, at a larger scale, specific subregions may decrease their activity to balance increased activity in a more remote subregion. In the context of an externally focused cognitive task, this might involve a reduction in activity in a specific PCC or inferior parietal lobe subregion, depending on which more remote brain regions increase their activity with the specific task. This would manifest itself as a spatially non-stationary DMN over time. This balancing could allow the brain to operate at high levels of activity without becoming unstable or inefficient. An analogy might be with a balloon that, as you squeeze in one part, automatically reshapes to compensate somewhere else (Fig. 5). Similarly, an increase in activity in one part of the brain to perform a task (e.g., external attentional focus) it is matched by a spatially linked deactivation in a nearby region that is not necessary for the task (Leech and others 2014).

Voltage imaging of mice performing simple tasks shows that, over time, activity flows into medial regions such as the retrosplenial cortex (associated with task deactivation in fMRI). This region seems to act as a “sink,” with activity flowing into, but not out of it (Mohajerani and others 2013). Similarly, resting state analyses of activity suggest that the PCC could also act as a sink, with its activity being driven by other regions, and that this sink function may be impaired following brain injury (Crone and others 2015). If this is the case, then such regions could be integral to a system that “mops up” over-excitation, allowing the brain to function at a high level of activity without becoming out of control. An analogy could be like the ballast on a boat that balances the distribution of forces and allows the boat to travel faster. Taking the analogy further, sailing boats can have active ballasts (the crew), which shift their position as the boat moves to counteract a broader range of forces and stop the boat from capsizing at even higher speeds. The relative modulation of echo subregions could similarly represent a shift in local dynamics in order to enable pronounced, but controlled, changes in macro-scale brain dynamics.

## Bringing It All Together

The explanations detailed above differ in important ways, and they may at first seem incompatible. However, it is plausible that the heterogeneous organization of transmodal cortex serves multiple, non–mutually exclusive roles. There are many examples in biology of phenomena having evolved for one purpose before being co-opted for another. For example, feathers evolved initially for some purpose other than flying, maybe to help with thermoregulation (e.g., Zhang and others 2010). Subsequently, exaptive evolutionary processes repurposed feathers for flying. Similarly, it is possible that much of the organization of the brain evolved to support much simpler sensory or motor control, rather than to specifically support high-level cognition. Systems that originally evolved to regulate neural dynamics or activity, could subsequently have been repurposed to perform more and more complex information processing. This is similar to the argument that language or reasoning is a new tool made out of old parts. From an evolutionary perspective, one approach is to ask how a system without the biological machinery to enable attentional selection would do it. If the starting point is a system where there are spontaneous neural dynamics, then to pay selective attention to a specific stimulus feature the system would have to modulate these existing dynamics to that end. Evolutionary pressures would therefore lead to more sophisticated and flexible control of these more basic systems, conducted by mechanisms which then become incorporated into the system. As an example, consider the DMN. It is present across many mammals (e.g. rats, monkeys, humans; Lu and others 2012; Mantini and others 2011) yet is associated with relatively complex (human specific) cognitive functions (e.g. moral judgments, theory of mind, long-term episodic memory; Buckner and others 2008). These seemingly conflicting findings could be because the DMN evolved initially to serve, for example, a basic homeostatic regulatory function: counterbalancing increases in activity in other brain regions during motor activity. Therefore, the DMN was more active when not performing externally focused tasks. This property may have meant that the DMN was the natural, and easiest system for evolutionary processes to “hack” when recruiting the neural resources to support more and more sophisticated internally focused cognitive abilities. More generally, it may be useful to view the brain as something that evolution has gradually tinkered with, such that the function-structure relationships reflect not just the behavior that is desired but also this “evolutionary history” itself.

## Summary/Conclusions

The findings of multiple, strongly discriminable neural subsignals within transmodal regions of the brain has important implications for neuroscience. First, when these subsignals are ignored (e.g., by taking the average signal across different echo regions), the remaining signal and FC pattern from a transmodal seed region will not be representative of its true complexity. Second, the existence of subsignals might explain why transmodal regions are implicated in multiple cognitive tasks. Cognitive tasks that recruit the same transmodal regions might be differentiated by considering that the LFA can display different patterns of modulation (in the same transmodal region) during different tasks. Third, although it has not been the focus of this review, different clinical conditions which are associated with the impairment of the same ICN (e.g. schizophrenia, depression, attention deficit/hyperactivity disorder, Alzheimer’s disease, or traumatic brain injury; Buckner and others 2008; Sharp and others 2014) might also be differentiated by considering the LFA. Finally, understanding the LFA may involve not only considering their functional role in cognition but also understanding how cognition emerges out of the brain as a biological organ. Processes such as the coordination of spontaneous dynamics and homeostatic regulation may play a role in explaining the complex organization of the brain.

## Supplementary Material

Supplementary material
